# The Nutritional Composition of Natural and Organic Branded Food Products: A Cross-Sectional Analysis of the Greek Foodscape

**DOI:** 10.3390/nu14040808

**Published:** 2022-02-14

**Authors:** Alexandra Katidi, Anthi Pavlopoulou, Antonis Vlassopoulos, Maria Kapsokefalou

**Affiliations:** Department of Food Science & Human Nutrition, Agricultural University of Athens, 11855 Athens, Greece; alexandra_katidi@aua.com (A.K.); anthipvl_3@yahoo.gr (A.P.); avlassopoulos@aua.gr (A.V.)

**Keywords:** natural, bio/organic, branded foods, packaged foods, claims, nutritional composition

## Abstract

Modern consumers turn to foods marketed as ‘natural/organic’ in their pursuit of healthier options. However, research that links such claims made on pack with improved nutritional composition is limited. The current analysis used data from all packaged foods available in the Hellenic Food Thesaurus *(n* = 4002), sold in Greece from 09/2020 to 01/2021, to map the prevalence of packaged foods sold under a ‘natural/organic’ claim and to compare their nutritional composition against food group matched conventional counterparts. Statistical analysis was carried out using IBM SPSS Statistics^®^. Overall, ‘free from’ was the most commonly used claim (12.3%), followed by ‘natural/pure’ (9.1%), ‘fresh’ (4.6%), and ‘bio/organic’ (3.3%). Statistically significant differences between the nutritional composition of natural/organic and conventional foods were only found in 5 out of the 13 food categories and in 9 out of 39 subcategories. Being labelled as natural/organic was linked to improved nutritional composition for prepared foods and yogurts, while for breakfast cereal, there was a mixed effect with lower carbohydrate content but higher energy and fat content. Jams labelled as natural/organic had higher energy and total sugar content. Overall, evidence of an association between being labelled as natural/organic and having an improved nutritional composition was extremely rare.

## 1. Introduction

Front-of-pack (FoP) visual and verbal claims play a critical role in capturing consumers’ attention, allowing them to quickly judge which product fits their diet and lifestyle needs [1,2]. Traditionally, claims made on pack aimed to link a food’s composition to specific health effects. However, lately the use of generic claims such as ‘healthy’ and ‘natural’ is increasingly seen on packaged foods, potentially as part of a general marketing strategy called the ‘health halo’ effect [3,4]. The intensity of usage of such terms and their ability to create confusion to the consumer has led official bodies such as the Food & Drug Administration (FDA) to issue definitions on the usage of the term ‘healthy’ [5,6,7]. Although the regulations of Nutrition and Health claims made on packaged products have been the topic of legislative and scientific discussion for nearly three decades [8,9], the proliferation of marketing strategies and venues, regulating an ever-expanding food labelling environment, requires constant vigilance [10] in order to ensure that any claim made on pack is not misleading and is substantiated by generally accepted scientific data [11].

In addition to functional foods (foods with specific health and nutrition claims), the past 5 years have seen an increase in consumer demand for natural and organic foods. According to legislation, a product is considered natural when ‘it contains no artificial ingredient or added color and is only minimally processed. Minimal processing means that the product was processed in a manner that does not fundamentally alter the product. The label must include a statement explaining the meaning of the term natural (such as “no artificial ingredients; minimally processed”)’. Similarly, organic food is any food produced without the use of artificial fertilizers (grown on soil that is restored only with organic fertilizers); without pesticides, growth regulators, antibiotics, hormones, and many other types of chemicals; and processed without the use of additives and chemical preservatives that are popular in the modern food industry [12,13].

Consumers are increasingly more aware and more willing to pay a price premium for organic or natural foods [14,15,16]. However, the increase in consumer demand for natural and organic products is only partially attributed to an increase in consumer awareness of such products [15,16,17]. Consumers are more likely to purchase organic products when they offer multiple benefits that cover a range of beliefs and attitudes. For example, previous research has linked the willingness to purchase organic foods with statements regarding improved animal welfare, a link to a product’s local origin, and its environmental impact, as well as the product’s nutritional value [14,18,19,20,21]. As far as nutritional value is concerned, consumers often assign higher nutritional value to both organic and natural foods, and this improved nutritional value is linked to a perceived improvement in the nutritional composition of foods labeled as organic or natural [9,19,22]. The importance of nutritional value as an element of organic/natural foods for the consumer is so great that in the absence of such a nutritional superiority, the added value assigned to the product diminishes greatly [23].

Despite consumer perceptions, research to date does not support the hypothesis that natural and/or organic foods have indeed an improved nutritional composition, except from small differences in selected nutrients [23]. Those differences are most commonly found in micronutrients and phytochemicals rather than macronutrients [24]. As micronutrient and phytochemical content are not part of the nutritional declaration made on pack, it is important to understand whether any consumer perception around the nutritional superiority of organic/natural foods could be readily justified on the basis of the information available to the consumer on pack. Unfortunately, research in this area is limited, as it requires access to large datasets with data on organic/natural foods and matched conventional counterparts to allow for head-to-head comparisons.

The Hellenic Food Thesaurus (HelTH) was established in 2019 as a tool to map and track packaged food product sold in Greece. HelTH tracks nutritional composition and all statements made on pack for ≈4000 products and it allows for cross-sectional analysis on the link between marketing statements and the nutritional composition of foods. For the current analysis, HelTH was used to quantify the prevalence and type of natural and organic/GMO-free claims on packaged foods sold in Greece, and we compared the nutritional composition of those products against category-matched conventional counterparts. The main research hypothesis was that a product’s natural or organic nature has limited impact on its final nutritional composition in macronutrients.

## 2. Materials and Methods

### 2.1. The HelTH Database

The Hellenic Food Thesaurus (HelTH), the Branded Food Composition Database of the Agricultural University of Athens, was used for data extraction (HelTH is a dynamic dataset that collects information on packaged foods sold in Greece, and it was first implemented in 2019. In brief, HelTH includes information on the nutritional composition of foods, any health and/or nutrition claims made on pack, and information on any other quality claims written on pack (environmental claims, logos, origin, etc.). A detailed description of the methodology and structure of HelTH has been published previously [25].

The first version of HelTH (11/2019) was used for the current analysis *(n* = 4002). Data were selected on the basis of the availability of nutritional composition data and the availability of data on specific marketing claims. The claims used for the current analysis were extracted from the variables including on-pack communication related to the naturality of products, their organic production, their relationship with the environment, and other social aspects.

All information around claims and the nutritional composition was taken from the packaging and entered into the database. Claims from all sides of the packaging were identified. Claims included both graphical indicators (logos) and text. All visible text was considered a claim, including brand slogans. Due to the fact that ‘natural’ claims—except biological/organic—have not been authorized or defined, data entered at these variables are varied, and further organization was needed. For the purpose of this study, a review of the claims entered at the database was conducted, and thus all variations in wording of naturality were identified (Table 1).

### 2.2. Data Selection and Cleaning

All data of the HelTH Branded Food Composition Database (BFCD) were checked and cleaned. In particular, duplicates of the same product, constituting part of an offer or discount multi-package, or by human error appearing twice at the online platform, were excluded (multi-pack items were deleted where the single item was also available).

**Figure 1:**
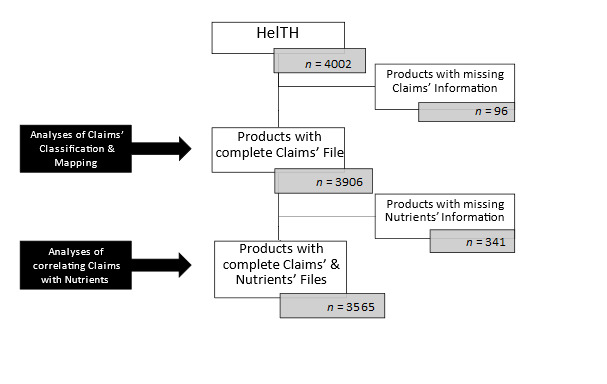
Flow chart of the products included in the analyses of this study. HelTH, Hellenic Food Thesaurus.

For the purpose of the study, products that did not contain any data about the claims (health, nutrition, organic, natural, etc.) *(n* = 96) were considered ineligible and were excluded. Furthermore, in analyses that included nutritional data, products missing nutrient information (e.g., energy, saturated fatty acids (SFA), sugar, sodium, protein, and fiber) due to the data collection and data entry methodology limitations (no access to photographs of all the sides of the products’ packaging or photographs not clear enough to copy data or researchers’ error) were also excluded *(n =* 341) (Figure 1).

### 2.3. Analysis

Statistical analysis was carried out using IBM SPSS Statistics^®^ (version 23, Northridge, CA, USA). Nutritional composition data were analyzed as continuous variables (content per 100 g or 100 mL of product). Data were tested for normality using the Kolmogorov-Smirnov test. None of the variables followed the normal distribution. Therefore, variables were expressed as median (interquartile range). Differences were tested using the Kruskal-Wallis non-parametric test for k independent samples. Between-group differences were tested using the Mann-Whitney U test for continuous variables. Statistical significance was set at 0.01% to adjust for multiple comparisons (Bonferroni correction).

## 3. Results

### 3.1. Categorization and Number of Claims

After processing the differences in wording and clustering their meanings, we classified claims for naturality and biological production into five groups. Table 2 provides an overview of the expressions of natural and other organic/genetically modified organisms (GMO)-free claims displayed on Greek foods. Claims for naturality were grouped into ‘free from’ claims (*n* = 471), ‘natural/pure’ (*n* = 349), or ‘fresh’ (*n* = 177), and other claims were categorized as a product being either biological/organic (*n =* 127) or GMO-free (*n* = 47). Other claim categories referring to the packaging: fresh pack (*n* = 20) and recyclable packaging (*n* = 428), as well as social claims: respect to the environment (*n* = 59), animal welfare (*n* = 46), sustainability *(n* = 45), and employee support (*n* = 34) were considered out of the scope for the present study and were excluded from the current analyses.

Overall, 22% (*n* = 853) of the products carried at least one claim for being natural, while less than 5% of the products (*n* = 172) carried other organic–GMO-free claims. The most prevalent claim was the absence of preservatives, found at about half (48.7%) of the products carrying a ‘natural’ claim, was followed by the ‘natural ingredients’ claim found at 22.3% of these products. The average number of ‘natural’ claims per product was 1.5, while nearly none (0.1%) of the products combined a bio/organic and ‘GMO-free’ claim. A total of 10.6% (*n* = 90) of the products bearing a ‘natural’ claim had a relevant logo on their packaging, while the majority of the products with an organic–GMO-free claim were accompanied by the relevant logo (aligned with legislator obligations) [26].

The prevalence of natural and organic–GMO-free claims was assessed overall and by food categories and subcategories. In total, 1171 food products (30.0%) carried claims on ‘naturality’, organic production, and/or absence of GMOs. The detailed distribution of those claims per food category and subcategory is presented at Table 3. More than a quarter of the ‘free from’ claims (28.9%) and ‘natural/pure’ claims (28.7%) were found in ‘the grain or grain product’ category, and most of them (44.1% and 44.0%, respectively) were in the ‘bread or similar product’ subcategory. ‘Fresh’ claims were more prevalent in the ‘milk, milk product, or substitute’ category. Bio/organic claims were found in 10 out of 13 food categories, with percentages varying from 0.6% to 8.2%, except for the ‘egg or egg product’ category, wherein more than one-third of the products (37.1%) were characterized as biological. The ‘GMO-free’ claim appeared on the packaging of 47 food products in total, belonging to specific food categories, the majority of them (59.6%) to the ‘milk, milk product, or substitute’ category.

### 3.2. Nutritional Composition of Products Bearing ‘Natural’ and Organic–GMO-Free Claims

As the distribution of claims on ‘naturality’, organic, and GMO-free labels were not evenly distributed across the food groups, it was paramount that any differences in the nutritional composition of natural, organic, and GMO-free foods were tested against conventional counterparts in the same food group. Analysis within the same food subcategory showed that differences between natural and/or organic–GMO-free and conventional foods were only found in 5 out of the 13 food categories and for specific claims (Table 4). The results of those five categories are described in detail below.

In the ‘milk, milk product, or milk substitute’ category, being labeled as fresh was associated with significantly lower carbohydrates and total sugar content (*p <* 0.001), but only for yogurts and not the remainder of the category.

In the ‘grain or grain product’ category, statistically significant differences could be found in the ‘cereal or cereal-like milling product or derivatives’ subcategory for total sugars (*p <* 0.001). In the ‘pasta or similar product’ subcategory, being labelled as organic was associated with lower protein content (*p <* 0.001). In the ‘breakfast cereals’ subcategory, differences were only found for products labelled as natural, and they all exhibited higher content of total fat, saturated fatty acids (SFA), carbohydrates, and energy (*p <* 0.001), while in the ‘fine bakery wares’ subcategory, products labelled as ‘free from’ had a higher total sugar content (*p <* 0.001).

In the ‘sugar or sugar product’ category, products in the ‘jam or marmalade’ subcategory showed statistically significantly higher energy, carbohydrate, and total sugar contents (*p* ≤ 0.001) when labelled as fresh compared to products without such a claim.

In the ‘beverage (non-milk)’ category, products belonging to the ‘juice or nectar’ subcategory bearing a natural claim presented themselves as significantly lower in carbohydrates and total sugars and higher in salt content (*p* < 0.001) than conventional products. On the contrary, ‘juices or nectars’ labelled as fresh had significantly higher protein content and significantly lower energy and salt content (*p* ≤ 0.001) than the conventional products. For this particular food category, it is important highlight that despite statistical significance, the absolute content in protein, total fats, and salt is very low, irrespective of any on pack claims.

Within the ‘miscellaneous food products’ category, ‘spice, condiment, or other ingredient’ labelled as ‘free from’ had significantly lower content of energy, carbohydrates, total sugars, salt, fiber, and protein (*p* < 0.001) than products without such claims. For the ‘prepared food products’ labelled as ‘free from’, they were associated with significantly lower in energy, total fat, saturated fat, carbohydrate, and salt content (*p* ≤ 0.001), whereas being labelled as ‘fresh’ was associated with significantly lower energy and salt content (*p* ≤ 0.001).

## 4. Discussion

The present study investigated the differences in the nutritional composition of foods with and without ‘natural’ and organic/GMO claims on packaged foods currently sold in Greece. Overall, approximately one-quarter (22.2%) of foods of the HelTH BFCD carried at least one natural claim, and 4.3% carried at least one organic/GMO claim. It appears that when foods carry claims, they tend to carry more than one, which is often the case for natural claims. Natural foods carry one to five claims on their packaging, while 90 of these products carried a logo also related to their naturalness. The prevalence of claims related to products’ naturality variates according to the food category (0.0–22.9%) and subcategory (0.0–63.0%) and the type of claim. Among the food products analyzed, only 127 were bio/organic, usually appearing with small percentages in most of the food categories, while ‘GMO-free’ claims were prevalent in only specific subcategories.

Overall, yogurts, spice/condiments, prepared foods, jams, and grains were the main categories in which carrying a claim around naturality or being organic was linked to any differences in the nutritional composition. Differences were more commonly seen for total or saturated fat content, followed by energy, total sugars, and salt. It is important to mention that differences were only seen in specific claim categories, even within the same food subcategory. Whether consumers differentiate between the perceived nutritional value of fresh, natural, and organic foods should be further studied.

These findings are particularly important for the discussion around the nutritional quality of products sold under labels such as natural, free from, organic, and GMO-free. According to our findings, such labels are rarely linked to better nutritional composition, despite common consumer beliefs. In fact, it is important to highlight that those findings would be very different if the statistical analysis was not performed in a food category matched manner.

Food category matching is extremely important if consumer protection from misleading marketing is at the heart of any analysis. As food categories have intrinsically different nutritional composition, analysis of the foodscape as a whole would be erroneous and would lead to misguided choices. Conversely, if the current analysis was performed without a food category matching the results would suggest that food products carrying claims for naturality and/or organic–GMO-free claims seem to have lower contents of energy and nutrients compared to their conventional counterparts (data not shown). These tantalizing findings, however, would be merely an artefact of different distribution among food categories rather than a true difference in head-to-head comparisons between identical foods. Even though, as mentioned earlier, nutrition and health-related claims are in the scope of being examined by the scientific community, there is a limited number of studies dealing with natural and other similar claims. According to a study published in 2003 [27] examining the nutrition and health-related claims used on packaged Australian foods, the ‘preservative-free’ claim was found on 20.1% of all products and was used on more than 40% of canned foods, chips, juices, meat substitutes, pretzels, and rice cakes. ‘No artificial colors’ was claimed on 17.6% of products, and ‘no artificial flavors’ on 14.8%; GMO-free was claimed on 1.7%, and bio/organic on 1.1% of all products. These results agree to some extent with ours, as the most prevalent claims were also—and in the same order—‘no preservatives’, ‘no artificial colorants’, and ‘no artificial flavors’. Organic claims (3.3%) were most common on egg or eggs products (37%), and GMO-free claims (1.2%) were also usual on milk and milk substitutes (4.0%) in both studies.

Although these claims from a technological standpoint are expected to have a minor impact on the macronutrient composition of a food product [19,22,23], consumers are generally willing to pay high price premiums for products with these attributes as they are perceived to have higher nutritional value [9]. Although some evidence exists to link consumption of organic foods to reduced risk of allergic disease and of overweight and obesity [28,29,30], this might not be explained via a difference in the macronutrient composition of those products. Our results agree with the Food Labelling of Italian Products (FLIP) Study [23], which indicated that, with few exceptions, organic labelled prepacked products were not characterized by a better nutritional composition than conventional ones. Bio/organic claims should not be interpreted by consumers as proxy of ‘healthier’ food than regular food when comparisons are made between identical food products. A previous systematic review in various food categories also highlighted differences in the micronutrient and phytochemical content of organic foods and not in macronutrients [30].

This is the first comprehensive study, to our knowledge, that deals with natural and organic–GMO-free claims, as well as assessing their prevalence in the Greek marketplace. Aside from providing the first overview and a baseline for the labelling indications in Greece, the present study offers a novel method of classifying natural and organic–GMO-free claims in which the different wording of the same claims was rigorously examined. It is hoped that the categorization developed will provide guidance for other researchers on replicating and advancing future claim prevalence studies, as this approach required more coordination efforts, but we believe that this was appropriate and would be the most suitable method for future similar research. Furthermore, this study assessed the nutritional composition of the products carrying natural and/or organic–GMO-free claims and indicates the necessity of stepping towards making research a useful source of information for future regulation, as it underlines the need of the development of a framework for the unregulated claims existing on food packaging and the need of a system to evaluate the suitability of foods for carrying them and to ensure consumers’ protection.

This study had also some limitations worth highlighting. The first is in regard to the lack of market share data and the shortcoming of evaluating the extent to which the HelTH BFCD covers the Greek pre-packaged food market. Secondly, the present study focused on the evaluation of nutritional composition only on the basis of mandatory information, which does not include other nutritional components, such as vitamins, minerals, and bioactive compounds. Moreover, it is important to note that, considering the Regulation 1169/2011 [31], nutrition declaration can be formulated either from direct analysis of food or from data extrapolated from reference databases of food composition, which do not take into account potential differences between natural, organic, and non-organic ingredients.

## 5. Conclusions

Foods labelled as natural and organic/GMO-free are rarely different to their food category-matched conventional counterparts in terms of nutritional composition. In our study, only 9 out of 39 food subcategories showed any evidence of such a difference in nutritional composition. The development of detailed food composition databases that combine labelled data such as organic, natural, and other claims with macro-nutrient, micro-nutrient, and phytochemical composition will be required to further explore potential differences in nutritional quality.

## Figures and Tables

**Table 1:** Variations in wording used for natural and other claims.

Free from/No/Without/Absence of	Natural/Pure	Fresh
Preservatives	Natural caffeine/vanilla from Madagascar/mastic oil from Chios	100% fresh apple/(squeezed) juice/(Greek) milk etc.
Additives	(Made with) natural flavor/flavorings	Fresh dough/milk/butter/Atlantic salmon/pork meat etc.
(Artificial) colorants/(food) color additives	Only natural flavor	85% from fresh fruit pieces
(Artificial) flavorings/flavor enhancers/added flavors	With natural flavorings of lemon/orange/lime etc.	Alive
Artificial sweeteners	With natural mineral water from Zagorochoria source	Fresh like the day it was caught
Pesticide residues/pesticides	100% extra virgin olive oil	Freshly ground in our mill
Artificial aromas	With extra virgin olive oil	Produced same day as milking/only with milk of the day
Chemical treatment	100% fillet/meat/juice/ground sesame etc.	Freshly trimmed
(Artificial) improvers	100% natural honey	From (100%) fresh (Greek) milk
Chemical fertilizers	100% from fruits and fruit juices	Made with fresh potatoes
	Natural fruit pieces	With 100% few hours’ Greek milk from Macedonia
	100% natural (product)	Fresh pack/pure pack
	Naturally brewed/sun ripened	
	Natural yeast	
	Natural sourdough	
	Natural sweeteners	
	Only natural ingredients	
	(All) natural ingredients	
	100% natural product	
	100% natural must	
	(Contains) natural sugars	
	Only natural sugars	
	Only with natural sugars occurring from fruits	
	Naturally occurring sodium/sugars	
	Naturally enriched with protein/vitamins/minerals	
	Natural source of (energy/protein/fiber/vitamins/minerals)	
	Farmer-owned	
	Your natural taste/naturally refreshing taste/naturally tasty/ Physical taste of/colors from nature	
	(With) genuine sesame/Florina peppers/authentic sea flavor	
	From 100% cow/goat/goat–sheep milk	
	Full of nature in every drop	
Organic–GMO-free claims	From pure ingredients	
Biological Bio Organic	From concentrated natural (orange/apple etc.) juice	
	From pure goat milk	
	Pure	

**Table 2:** Classification and number of natural and environment-related claims existing in the packaging of the branded food products of the HelTH BFCD.

Products with Claims			Claims Per Product *n* (%)				
		*n* (%)	1	2	3	4	5
Claims about naturality		853 (22.2)	536(14.0)	220(5.7)	65(1.7)	23(0.6)	1(0.0)
Free from		471 (12.3)	299 (7.8)	123 (3.2)	49 (1.3)		
	No preservatives	415 (10.8)					
	No artificial colorants	164 (4.3)					
	No artificial flavorings/ flavor enhancers	83 (2.2)					
	No additives	16 (0.4)					
	No pesticides	6 (0.2)					
	No chemical fertilizers/processing	5 (0.1)					
	No improvers	3 (0.1)					
Natural/Pure		349 (9.1)	301 (7.8)	46 (1.2)	2 (0.1)		
	Natural ingredients	190 (4.9)					
	Natural product	88 (2.3)					
	Natural taste/flavor/color	81 (2.1)					
	Natural source of nutrients	29 (0.8)					
Fresh		157 (4.6)	157 (100)		-		
	Fresh ingredients	93 (2.4)					
	Fresh product	64 (1.7)					
Other claims		172 (4.3)	169 (4.2)	3 (0.1)			
Bio-Organic		127 (3.3)					
GMO-free		47 (1.2)					

**Table 3:** Prevalence of natural and organic–GMO-free claims through the food categories and subcategories of the HelTH BFCD.

	Free From	Natural/Pure	Fresh	Bio-Organic	GMO-Free
Food Categories and Subcategories	*n* (%)	*n* (%)	*n* (%)	*n* (%)	*n* (%)
Milk, milk product, or milk substitute (*n* = 693)	69 (10.0)	62 (8.9)	64 (9.2)	29 (4.2)	28 (4.0)
Milk (*n* = 175)	8 (4.6)	22 (12.6)	0 (0.0)	12 (6.9)	10 (5.7)
Yogurt (*n* = 172)	30 (17.4)	16 (9.3)	46 (26.7)	5 (2.9)	8 (4.7)
Cheese (*n* = 212)	10 (4.7)	18 (8.5)	17 (8.0)	6 (2.8)	0 (0.0)
Cream (*n* = 42)	12 (28.6)	0 (0.0)	11 (26.2)	0 (0.0)	0 (0.0)
Imitation milk products (*n* = 52)	8 (15.4)	4 (7.7)	0 (0.0)	6 (11.5)	10 (19.2)
Frozen dairy desserts (*n* = 40)	1 (2.5)	2 (5.0)	0 (0.0)	0 (0.0)	0 (0.0)
Egg or egg product (*n* = 35)	4 (11.4)	0 (0.0)	0 (0.0)	13 (37.1)	7 (20.0)
Fresh or processed egg (*n* = 35)	4 (11.4)	0 (0.0)	0 (0.0)	13 (37.1)	7 (20.0)
Meat or meat product (*n* = 156)	1 (0.6)	4 (2.6)	13 (8.3)	1 (0.6)	0 (0.0)
Preserved meat products (*n*= 89)	0 (0.0)	1 (1.1)	6 (6.7)	0 (0.0)	0 (0.0)
Sausage or similar meat products (*n* = 38)	1 (2.6)	3 (7.9)	7 (18.4)	1 (2.6)	0 (0.0)
Meat dish (*n* = 26)	0 (0.0)	0 (0.0)	0 (0.0)	0 (0.0)	0 (0.0)
Seafood or related product (*n* = 80)	6 (7.5)	8 (10.0)	1 (1.3)	0 (0.0)	0 (0.0)
Seafood products (*n* = 80)	6 (7.5)	8 (10.0)	1 (1.3)	0 (0.0)	0 (0.0)
Fat or oil (*n* = 81)	7 (8.6)	10 (12.3)	0 (0.0)	4 (4.9)	0 (0.0)
Vegetable fat or oil (*n* = 8)	0 (0.0)	1 (12.5)	0 (0.0)	0 (0.0)	0 (0.0)
Margarine or fat from mixed origin (*n* = 39)	5 (12.8)	1 (2.6)	0 (0.0)	1 (2.6)	0 (0.0)
Butter or other animal fat (*n* = 34)	2 (5.9)	8 (23.5)	0 (0.0)	3 (8.8)	0 (0.0)
Grain or grain product (*n* = 1129)	136 (12.7)	100 (9.3)	34 (2.2)	29 (2.7)	1 (0.1)
Cereal or similar milling product (*n* = 51)	0 (0.0)	0 (0.0)	9 (17.6)	0 (0.0)	0 (0.0)
Rice or similar grain (*n* = 89)	2 (2.2)	0 (0.0)	0 (0.0)	7 (7.9)	0 (0.0)
Pasta or similar product (*n* = 203)	0 (0.0)	0 (0.0)	0 (0.0)	11 (5.4)	0 (0.0)
Breakfast cereal (*n* = 150)	30 (30.3)	23 (23.2)	0 (0.0)	2 (2.0)	0 (0.0)
Bread or similar product (*n* = 259)	60 (23.2)	44 (17.0)	23 (8.9)	8 (3.1)	1 (0.4)
Fine bakery ware (*n* = 289)	19 (6.6)	13 (4.5)	2 (0.7)	1 (0.3)	0 (0.0)
Savory cereal dish (*n* = 83)	25 (30.1)	20 (24.1)	0 (0.0)	0 (0.0)	0 (0.0)
Nut, seed, or kernel (*n* = 131)	15 (11.5)	30 (22.9)	0 (0.0)	7 (5.3)	0 (0.0)
Nuts (*n* = 69)	0 (0.0)	0 (0.0)	0 (0.0)	1 (1.4)	0 (0.0)
Seeds or kernels (*n* = 35)	6 (17.1)	13 (37.1)	0 (0.0)	4 (11.4)	0 (0.0)
Nut/seed product (*n* = 27)	9 (33.3)	17 (63.0)	0 (0.0)	2 (7.4)	0 (0.0)
Vegetable or vegetable product (*n* = 244)	45 (18.4)	4 (1.6)	8 (3.3)	20 (8.2)	0 (0.0)
Vegetable (excluding potato) (*n* = 172)	40 (23.3)	4 (2.3)	0 (0.0)	16 (9.3)	0 (0.0)
Starchy root or potato (*n* = 21)	5 (23.8)	0 (0.0)	8 (38.1)	0 (0.0)	0 (0.0)
Pulse or pulse products (*n* = 51)	0 (0.0)	0 (0.0)	0 (0.0)	4 (7.8)	0 (0.0)
Fruit or fruit product (*n* = 45)	2 (4.4)	0 (0.0)	0 (0.0)	0 (0.0)	0 (0.0)
Processed fruit product (*n* = 45)	2 (4.4)	0 (0.0)	0 (0.0)	0 (0.0)	0 (0.0)
Sugar or sugar product (*n* = 359)	55 (15.3)	23 (6.4)	12 (3.3)	10 (2.8)	5 (1.4)
Jam or marmalade (*n*= 83)	23 (27.7)	11 (13.3)	9 (10.8)	2 (2.4)	0 (0.0)
Non-chocolate confectionary or other sugar product (*n* = 68)	26 (38.2)	3 (4.4)	0 (0.0)	4 (5.9)	3 (4.4)
Chocolate (*n* = 208)	6 (2.9)	9 (4.3)	3 (1.4)	4 (1.9)	2 (1.0)
Beverage (non-milk) (*n* = 412)	62 (15.5)	88 (21.9)	25 (6.2)	5 (1.3)	3 (0.7)
Juice or nectar (*n* = 165)	44 (26.7)	35 (21.2)	25 (15.2)	5 (3.0)	3 (1.8)
Non-alcoholic beverage (*n* = 247)	18 (7.6)	53 (22.5)	0 (0.0)	0 (0.0)	0 (0.0)
Miscellaneous food products (*n* = 457)	61 (13.3)	17 (3.7)	9 (2.0)	9 (2.0)	2 (0.4)
Spice, condiment, or other ingredient (*n* = 283)	36 (12.7)	5 (1.8)	0 (0.0)	4 (1.4)	0 (0.0)
Prepared food product (*n* = 174)	25 (14.4)	12 (6.9)	9 (5.2)	5 (2.9)	2 (1.1)
Ready meals (*n* = 84)	8 (9.5)	3 (3.6)	1 (1.2)	0 (0.0)	1 (1.2)
Ready-to-eat meals (*n* = 43)	0 (0.0)	1 (2.3)	0 (0.0)	0 (0.0)	0 (0.0)
Frozen, semi-ready meals (*n* = 41)	8 (19.5)	2 (4.9)	1 (2.4)	0 (0.0)	1 (2.4)
Total (*n* = 3906)	471 (12.3)	349 (9.1)	177 (4.6)	127 (0.3)	47 (1.2)

**Table 4:** Nutritional comparison of products bearing natural or bio/GMO claims and conventional products in the subcategories that presented statistically significant differences.

Food Subcategories		Energy (kcal)	Protein(g)	Total Fat (g)	Saturated Fat (g)	Carbohydrates (g)	Sugars (g)	Fiber (g)	Salt (g)
Yogurt	Fresh (*n* = 46)	74.5 (71.0, 96.0)	7.9 (5.0, 9.1)	2.0 (1.7, 5.0)	1.3 (1.2, 3.6)	4.5 (3.8, 7.4)	4.2 (3.8, 7.3)	0.0 (0.0, 0.0)	0.12 (0.10, 0.17)
	No claim (*n* = 114)	80.0 (66.0, 102.0)	5.6 (4.6, 7.2)	2.0 (1.6, 3.9)	1.3 (1.0, 2.6)	7.5 (4.5, 13.8)	6.4 (4.2, 11.0)	0.2 (0.0, 0.8)	0.11 (0.10, 0.15)
	*p*-value	0.885	0.02	0.144	0.083	**<0.001**	**<0.001**	0.180	0.360
Cereal or cereal-like milling product	Fresh (*n* = 9)	342.0 (254.0, 352.5)	8.2 (7.7, 9.5)	15.7 (2.6, 18.4)	7.6 (1.1, 9.9)	40.8 (37.2, 46.7)	6.0 (5.8, 11.7)	1.7 (1.5, 2.0)	1.76 (1.58, 1.91)
	No claim (*n* = 29)	304.0 (274.0, 378.5)	7.5 (6.7, 8.4)	5.0 (3.9, 22.5)	1.4 (0.6, 5.2)	41.3 (36.6, 57.5)	1.6 (0.2, 3.2)	1.9 (1.5, 3.7)	1.20 (0.80, 1.30)
	*p*-value	0.460	0.128	0.548	0.030	0.595	**<0.001**	0.285	0.002
Pasta or similar product	Organic (*n* = 189)	358.0 (353.0, 358.0)	11.0 (11.0, 11.0)	1.5 (1.5, 2.0)	0.2 (0.2, 0.6)	75.0 (69.0, 75.0)	1.8 (1.8, 3.2)	3.0 (3.0, 4.1)	0.01 (0.00, 0.01)
	No claim (*n* = 11)	355.0 (352.0, 359.0)	12.0 (12.0, 13.0)	2.0 (1.5, 2.5)	0.4 (0.3, 0.5)	71.7 (67.7, 72.1)	3.2 (3.0, 3.8)	3.0 (2.9, 3.3)	0.03 (0.01, 0.07)
	*p*-value	0.417	**<0.001**	0.586	0.115	0.144	0.008	0.510	0.023
Breakfast cereal	Natural/pure (*n* = 23)	453.0 (417.0, 495.0)	8.0 (6.0, 14.0)	18.5 (15.0, 27.6)	5.0 (2.7, 8.0)	56.0 (48.4, 67.0)	21.4 (15.5, 26.5)	7.1 (6.1, 11.0)	0.50 (0.23, 0.97)
	No claim (*n* = 131)	393.0 (376.0, 430.0)	8.1 (6.8, 9.0)	7.5 (4.0, 15.0)	2.4 (1.0, 4.5)	67.5 (62.0, 74.0)	23.1 (15.8, 26.9)	6.9 (5.0, 8.5)	0.60 (0.29, 0.80)
	*p*-value	**<0.001**	0.657	**<0.001**	**<0.001**	**<0.001**	0.677	0.134	0.896
Fine bakery ware	Free from (*n* = 14)	468.0 (282.8, 510.0)	7.9 (6.7, 9.2)	26.0 (14.0, 27.0)	6.6 (3.7, 10.4)	55.3 (16.3, 17.1)	16.3 (13.4, 17.1)	3.3 (1.4, 4.8)	0.61 (0.33, 1.82)
	No claim (*n* = 228)	477.0 (438.0, 499.0)	6.6 (5.4, 8.0)	21.0 (17.0, 25.0)	9.8 (6.7, 13.0)	61.0 (54.3, 67.0)	26.0 (19.0, 35.0)	2.5 (1.8, 3.4)	0.50 (0.30, 0.75)
	*p*-value	0.556	0.012	0.451	0.014	0.005	**<0.001**	0.573	0.107
Jam or marmalade	Fresh (*n* = 9)	258.0 (255.5, 263.0)	0.7 (0.4, 1.6)	0.3 (0.2, 0.3)	0.0 (0.0, 0.0)	64.0 (62.5, 65.0)	62.5 (60.0, 63.2)	1.7 (1.4, 2.5)	0.00 (0.00, 0.00)
	No claim (*n* = 51)	237.0 (164.0, 244.0)	0.5 (0.4, 0.8)	0.1 (0.0, 0.3)	0.0 (0.0, 0.0)	58.0 (40.0, 60.0)	55.0 (22.0, 58.0)	1.7 (1.1, 2.3)	0.04 (0.01, 0.06)
	*p*-value	**<0.001**	0.447	0.031	0.029	**<0.001**	**<0.001**	0.607	0.007
Juice or nectar	Free from (*n* = 44)	47.9 (41.0, 51.0)	0.3 (0.2, 0.5)	0.0 (0.0, 0.2)	0.0 (0.0, 0.0)	11.5 (10.1, 12.2)	10.5 (9.1, 11.8)	0.4 (0.2, 0.6)	0.00 (0.00, 0.02)
	No claim (*n* = 118)	49.0 (46.0, 52.0)	0.4 (0.2, 0.6)	0.0 (0.0, 0.1)	0.0 (0.0, 0.0)	11.8 (11.0, 12.3)	11.4 (10.4, 12.0)	0.5 (0.4, 0.6)	0.00 (0.00, 0.01)
	*p*-value	0.127	0.169	0.214	**<0.001**	0.109	0.024	0.200	0.224
	Natural/pure (*n* = 35)	46.0 (44.0, 48.4)	0.4 (0.2, 0.4)	0.0 (0.0, 0.1)	0.0 (0.0, 0.0)	11.0 (10.7, 11,7)	10.4 (9.5, 10.9)	0.5 (0.3, 0.6)	0.03 (0.01, 0.03)
	No claim (*n* = 127)	49.0 (45.8, 53.0)	0.4 (0.2, 0.5)	0.0 (0.0, 0.1)	0.0 (0.0, 0.0)	12.0 (12.8, 11.0)	11.5 (10.4, 12.2)	0.5 (0.4, 0.5)	0.00 (0.00, 0.01)
	*p*-value	**<0.001**	0.815	0.180	**<0.001**	**<0.001**	**<0.001**	0.471	**<0.001**
	Fresh (*n* = 25)	48.0 (46.5, 50.5)	0.6 (0.5, 0.7)	0.0 (0.0, 0.1)	0.0 (0.0, 0.0)	11.2 (11.0, 12.0)	10.5 (10.0, 11.7)	0.3 (0.1, 1.2)	0.00 (0.00, 0.00)
	No claim (*n* = 137)	49.0 (45.0, 52.0)	0.3 (0.2, 0.5)	0.0 (0.0, 0.1)	0.0 (0.0, 0.0)	11.8 (10.9, 12.4)	11.2 (10.4, 12.0)	0.5 (0.4, 0.6)	0.00 (0.00, 0.02)
	*p*-value	0.952	**<0.001**	0.821	0.324	0.280	0.117	0.613	**<0.001**
Spice, condiments	Free from (*n* = 36)	6.0 (5.0, 47.5)	0.5 (0.5, 0.5)	0.5 (0.5, 0.5)	0.2 (0.2, 0.3)	0.5 (0.5, 1.4)	0.5 (0.5, 1.2)	0.5 (0.5, 0.5)	1.04 (0.88, 1.20)
	No claim (*n* = 211)	149 (93.0, 334.4)	2.8 (1.2, 5.3)	4.6 (0.5, 18.9)	0.7 (0.1, 4.7)	15.0 (6.5, 24.4)	6.2 (3.9, 21.6)	1.5 (1.0, 2.7)	1.80 (1.00, 3.45)
	*p*-value	**<0.001**	**<0.001**	**<0.001**	0.027	**<0.001**	**<0.001**	**<0.001**	**<0.001**
Prepared food products	Free from (*n* = 22)	116.0 (27.3, 293.8)	1.1 (0.7, 6.3)	1.3 (0.5, 3.8)	0.3 (0.1, 1.2)	5.7 (4.4, 37.8)	2.5 (0.8, 8.0)	0.5 (0.5, 1.7)	0.89 (0.30, 1.03)
	No claim (*n* = 131)	365.0 (247.0, 505.0)	3.8 (2.1, 7.0)	23.4 (6.0, 30.4)	4.3 (1.7, 6.3)	45.8 (8.4, 57.7)	1.7 (0.6, 3.7)	2.0 (1.0, 5.0)	1.50 (0.90, 1.83)
	*p*-value	**<0.001**	0.015	**<0.001**	**<0.001**	**<0.001**	0.176	0.004	**<0.001**
	Fresh (*n* = 8)	123.0 (95.3, 130.5)	3.0 (2.6, 3.1)	4.0 (3.1, 4.8)	2.6 (1.9, 5.9)	18.3 (14.8, 21.7)	8.1 (1.7, 16.0)	0.1 (0.0, 0.1)	0.16 (0.10, 0.23)
	No claim (*n* = 145)	357.0 (214.0, 503.5)	3.7 (1.5, 7.0)	21.3 (3.6, 30.4)	3.7 (1.0, 5.5)	40.1 (7.2, 57.4)	1.7 (0.6, 4.0)	1.9 (0.8, 5.0)	1.40 (0.88, 1.80)
	*p*-value	**<0.001**	0.283	0.035	0.960	0.431	0.089	0.002	**<0.001**

Values represent median (Q1, Q3). All statistical analyses were carried out with the Mann-Whitney U test. Bold highlights statistically significant values (*p* ≤ 0.001).

## Data Availability

Not Applicable.
